# Continuous subcutaneous insulin infusion therapy is associated with reduced retinopathy progression compared with multiple daily injections of insulin

**DOI:** 10.1007/s00125-021-05456-w

**Published:** 2021-05-08

**Authors:** Laura J. Reid, Fraser W. Gibb, Helen Colhoun, Sarah H. Wild, Mark W. J. Strachan, Karen Madill, Baljean Dhillon, Shareen Forbes

**Affiliations:** 1 Edinburgh Centre for Endocrinology and Diabetes, NHS Lothian, Edinburgh, UK; 2 BHF Centre for Cardiovascular Science, Queen’s Medical Research Institute, University of Edinburgh, Edinburgh, UK; 3 Institute of Genetics and Molecular Medicine, University of Edinburgh, Edinburgh, UK; 4 Usher Institute, University of Edinburgh, Edinburgh, UK; 5 Princess Alexandra Eye Pavilion, NHS Lothian, Edinburgh, UK; 6 Centre for Clinical Brain Sciences, School of Clinical Sciences, College of Medicine and Veterinary Medicine, University of Edinburgh, Edinburgh, UK

**Keywords:** Clinical diabetes, Clinical science, Insulin therapy, Microvascular complications, Retinopathy

## Abstract

**Aims/hypothesis:**

We aimed to compare diabetic retinopathy outcomes in people with type 1 diabetes following introduction of continuous subcutaneous insulin infusion (CSII) therapy with outcomes in people receiving continuing therapy with multiple daily insulin injections (MDI).

**Methods:**

This is a retrospective cohort study using the Scottish Care Information – Diabetes database for retinal screening outcomes and HbA_1c_ changes in 204 adults commenced on CSII therapy between 2013 and 2016, and 211 adults eligible for CSII during the same period but who continued on MDI therapy. Diabetic retinopathy progression (time to minimum one-grade worsening in diabetic retinopathy from baseline grading) was plotted for CSII and MDI cohorts using Kaplan–Meier curves, and outcomes were compared using multivariate Cox regression analysis adjusting for age, sex, baseline HbA_1c_, blood pressure, cholesterol, smoking status and socioeconomic quintile. Impact of baseline HbA_1c_ and change in HbA_1c_ on diabetic retinopathy progression was assessed within CSII and MDI cohorts.

**Results:**

CSII participants were significantly younger, were from less socially deprived areas, and had lower HbA_1c_ and higher diastolic BP at baseline. There was a larger reduction in HbA_1c_ at 1 year in those on CSII vs MDI (−6 mmol/mol [−0.6%] vs −2 mmol/mol [−0.2%], *p* < 0.01). Diabetic retinopathy progression occurred in a smaller proportion of adults following commencement of CSII vs continued MDI therapy over mean 2.3 year follow-up (26.5% vs 18.6%, *p* = 0.0097). High baseline HbA_1c_ (75 mmol/mol [9%]) was associated with diabetic retinopathy progression in the MDI group (*p* = 0.0049) but not the CSII group (*p* = 0.93). Change in HbA_1c_ at follow-up, irrespective of baseline glycaemic status, did not significantly affect diabetic retinopathy progression in either group.

**Conclusions/interpretation:**

CSII was associated with reduced diabetic retinopathy progression compared with continued MDI therapy, and may be protective against diabetic retinopathy progression for those with high baseline HbA_1c_. Progression of diabetic retinopathy over 3 years was not associated with a change in HbA_1c_.

**Graphical abstract:**

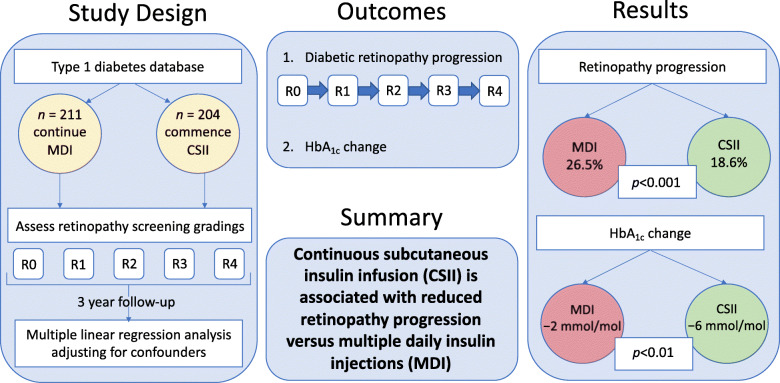

**Supplementary Information:**

The online version contains peer-reviewed but unedited supplementary material available at 10.1007/s00125-021-05456-w.

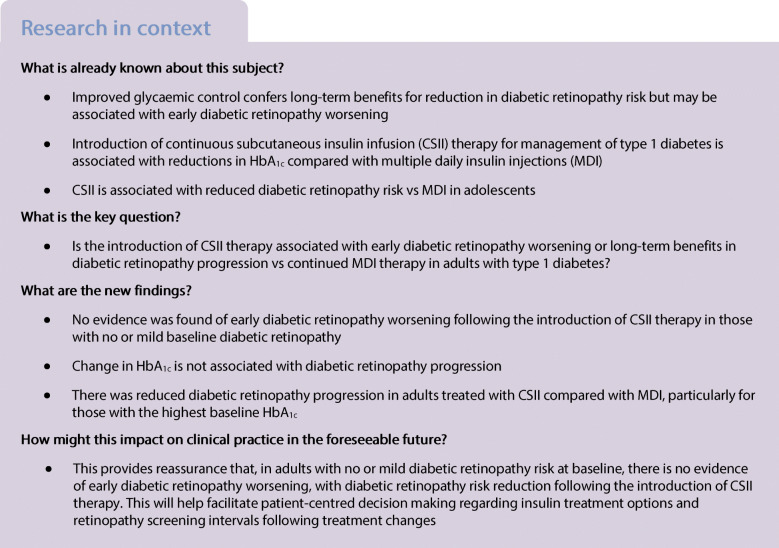



## Introduction

Diabetic retinopathy is one of the leading causes of blindness worldwide [1], and prevalence rises with age [2]. It has been well established, following the landmark DCCT, that good glycaemic control confers long-term benefits to reduce the risk of developing diabetic retinopathy [3]. However, concerns remain that rapid improvements in blood glucose level precipitate transient worsening of diabetic retinopathy [4], and many guidelines recommend controlled improvements in glycaemic targets with increased monitoring for retinopathy in the initial stages when treatment is altered [5].

In order to optimise glycaemic targets, insulin is typically delivered either via multiple daily insulin injections (MDI) or by continuous subcutaneous insulin infusion (CSII). A recent Cochrane review suggested that CSII is associated with a small but significant improvement in glycaemic control vs MDI therapy (0.3% absolute reduction in HbA_1c_) [6]. However, relatively few studies have evaluated whether treatment with CSII confers any benefits over MDI in reducing long-term diabetic retinopathy risk in an adult population, or whether there is any increased risk of early diabetic retinopathy worsening following a change in treatment from MDI to CSII.

Early studies assessing diabetic retinopathy progression following the introduction of CSII typically showed no improvement or deterioration of diabetic retinopathy with CSII [7, 8]. However, study numbers were small, participants were selected for CSII due to poor glycaemic control on conventional therapy and suitable comparator groups were not used. In addition, early insulin pump systems used in many previous study populations were more cumbersome as they did not have the ability to pre-programme variable basal rates and lacked safety alarms to notify users about infusion problems. A more recent study in adolescents suggests that CSII is associated with lower rates of retinopathy than MDI [9].

Our main aim in this study was to investigate retinopathy outcomes in adults with type 1 diabetes following the introduction of CSII therapy compared with those who continued on therapy with MDI using a robust clinical database system.

## Methods

### Study participants

Participants were identified from three diabetes centres within NHS Lothian, Scotland, using the Scottish Care Information – Diabetes (SCI-Diabetes) database, which links clinical information for all registered people with diabetes in Scotland using electronic care records in primary care, hospitals and pharmacies.

Diabetes records were reviewed for all participants with type 1 diabetes who were commenced on CSII therapy between 1 January 2013 and 1 December 2016 (*N* = 293). Retinal data collected during annual retinopathy screening for these participants were reviewed from 1 January 2011 to 1 December 2018. Within NHS Lothian, a 5 day structured education course on carbohydrate counting and diabetes management is recommended prior to referral for CSII therapy. To identify a control MDI group who had received a similar level of diabetes education to the CSII group, people who completed this course at a similar time to the CSII participants, but who remained on MDI therapy, were recruited for comparison (*N* = 277).

People were included if they were aged >16 years; had documented type 1 diabetes, defined as a clinical diagnosis of type 1 diabetes with no evidence in the historical record of >6 months between diagnosis and insulin requirement; and had no history of use of oral hypoglycaemic drug treatment other than adjuvant metformin. All participants were on treatment with either CSII or MDI therapy and were participating in the Scottish diabetes retinopathy screening programme during the study period. People were excluded if the date of commencing CSII therapy or completion of diabetes education could not be verified; if baseline or follow-up retinal images had not been taken, or those taken were deemed ungradable for both eyes; if they had been suspended from the retinal screening programme for assessment and potential treatment at ophthalmology clinics; or if they had the maximum severity retinopathy grading (R4) at baseline and could not therefore ‘progress’ to a higher diabetic retinopathy severity grade.

The study was approved by the South East Scotland Ethics Committee and NHS Lothian Caldicott Guardian, and was conducted according to the principles of the Declaration of Helsinki.

### Study design

This was a retrospective cohort study using routine clinical and diabetes retinal screening data. Data were extracted from SCI-Diabetes, including up-to-date information on retinal screening records, HbA_1c_, mode of current insulin therapy and diabetes education history, and anthropometric, metabolic and demographic data (weight, BMI, blood pressure, cholesterol, smoking history and an area-based measure of socioeconomic deprivation).

### Study time

Study entry (time = 0) for the CSII group was the date of commencing CSII therapy. Mean lag time from completing the recommended structured diabetes education course to commencing CSII therapy was 945 days (median 841 days, IQR 333–1555 days). Study entry for the MDI group was calculated as 945 days from the date of completion of the diabetes education course to give an equivalent entry time to the CSII group using the mean lag time from education to CSII commencement. Secondary analyses calculating study entry as 841 days from the date of completion of the diabetes education course were also completed using the median lag time from education to CSII commencement, and results for these analyses can be found in the electronic supplementary material (ESM). Study exit for CSII and MDI groups was taken as the earliest occurring of any of the following: a retinal event, emigration from Scotland, death or the end of the study period on 1 December 2018.

### Retinopathy assessment

Diabetic retinopathy assessment was performed as part of the Scottish diabetes retinal screening programme. A single macula-centred photograph is taken for each eye every 6 to 12 months and retinopathy grading assessed using the Scottish Diabetic Retinopathy Grading Scheme (SDRGS) [10] by experienced SDRGS-qualified graders. Severity of diabetic retinopathy was classified as: 0, no diabetic retinopathy; 1, mild background diabetic retinopathy; 2, moderate observable diabetic retinopathy; 3, referable diabetic retinopathy; or 4, proliferative diabetic retinopathy [10]. Repeatability of image grading was not assessed; however, these are the same data that are used to inform clinical decision making for further retinal assessment. Images were also assigned a maculopathy grading based on whether markers of macular oedema were present. For accurate assessment for the presence of macular oedema, 3D imaging, usually using ocular coherence tomography (OCT), is required as false positives are common from 2D fundus images. As such, this study focused on retinopathy gradings rather than maculopathy gradings.

As retinopathy gradings were available for both eyes, the more severe grading was used as the baseline grading.

The diabetic retinopathy screening assessment immediately prior to the study entry date was taken as the baseline grading (median 71 [IQR 18–243] days prior to study entry date).

Retinopathy progression was defined as a minimum one-grade worsening in either eye from the baseline grading.

### Assessment of glycaemic control by HbA_1c_

For each participant, HbA_1c_ data were extracted from the SCI-Diabetes database. Timings of baseline and follow-up HbA_1c_ values relative to the time of study entry were variable. The HbA_1c_ value immediately prior to the study entry date was taken as the baseline HbA_1c_ (median 75 [IQR 27–172] days prior to study entry date). Subsequent follow-up HbA_1c_ readings for each individual were identified for each subsequent 6 month period where available. Where multiple HbA_1c_ readings had been taken within a 6 month period, the HbA_1c_ value closest to, but not exceeding, the 6 month timepoint was used as the HbA_1c_ value for that interval. Mean study HbA_1c_ over 3 year follow-up was estimated by calculating the mean HbA_1c_ from the 6-monthly values for each individual. The HbA_1c_ at approximately 1 year post study entry was also recorded, with a range of 8 to 20 months from study entry accepted as the 1 year HbA_1c_ value in cases where 12 month values were not available. Study exit HbA_1c_ was recorded as the last available HbA_1c_ value recorded after 20 months from study entry.

### Statistical analyses

Baseline variables including age, sex, diabetes duration, baseline HbA_1c_, socioeconomic status (assessed using quintile of Scottish Index of Multiple Deprivation [SIMD] score [11]) and diastolic BP were summarised as median with IQR for continuous data and as percentage for categorical data. In the CSII vs MDI groups, baseline demographic and metabolic data were compared using the unpaired *t* test for continuous normally distributed data and χ^2^ test or Fisher’s exact test for categorical data. Kaplan–Meier plots were used to assess time to an event corresponding to retinopathy progression or exit from the study for another reason (death, emigration, end of study period) in CSII and MDI groups, with calculation of statistical differences between groups using the logrank test. Groups were not matched, but characteristics were similar between the groups and potential confounders were included as covariates in Cox proportional hazard analyses. Univariate and multivariate Cox proportional hazards analyses were performed using the following covariates: CSII or MDI treatment group, age, sex, diabetes duration, baseline HbA_1c_, systolic BP, diastolic BP, cholesterol, creatinine, SIMD, smoking status and baseline diabetic retinopathy grading.

To assess the impact of baseline, mean study, 1 year and study exit HbA_1c_, participants were stratified using HbA_1c_ values (<58 mmol/mol [<7.5%]), 58–75 mmol/mol [7.5–9%] and >75 mmol/mol [>9%]) and compared using Kaplan–Meier plots as above. Similarly, to assess the impact of the change in HbA_1c_ at 1 year, participants were stratified using change in HbA_1c_ value (>−5 mmol [>−0.5%], −5 to 5 mmol/mol [−0.5 to 0.5%] and >5 mmol/mol [>0.5%] change) and compared using Kaplan–Meier plots as above.

Statistical significance was assumed for *p* < 0.05. Statistical analyses were completed using R version 3.4.1 (https://www.R-project.org/.)

## Results

### Study populations

**Fig. 1:**
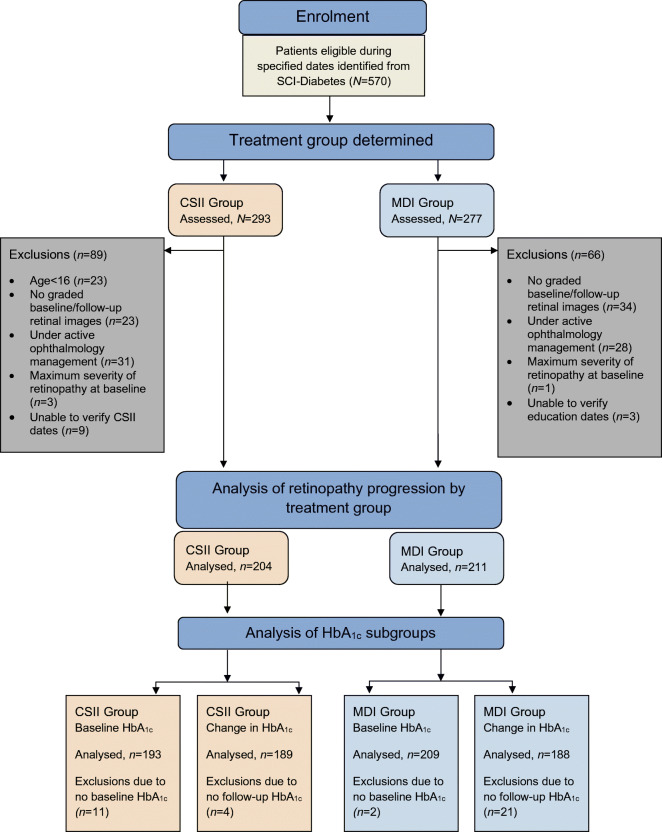
Flow chart showing number of people assessed and analysed for CSII and MDI groups and exclusions

Between 1 January 2013 and 1 December 2016, 293 people with type 1 diabetes who commenced CSII therapy were identified. Of these, 89 were excluded: 23 were aged <16 years at commencement of CSII, 23 had no available baseline and/or follow-up graded retinal screening results, 31 had been excluded from screening for further ophthalmology assessment or treatment, three had the maximum severity retinopathy grading at baseline and for a further nine we were not able to verify if the dates for commencing CSII were correct. The remaining 204 were included in the analysis (Fig. 1).

We identified 277 MDI control participants who had completed structured diabetes education at a similar time to the CSII cohort but who did not proceed to CSII therapy, largely due to patient preference. Of these, 66 were excluded: 34 had no available baseline and/or follow-up graded retinal screening results, 28 had been excluded from screening for further ophthalmology assessment or treatment, one had the maximum severity grading at baseline and for a further three we were not able to verify if the dates for diabetes education were correct. The remaining 211 were included in the analysis (Fig. 1).

Baseline demographic and metabolic data are shown in Table 1. CSII participants were significantly younger, with earlier age of diabetes diagnosis, lower baseline HbA_1c_, higher diastolic BP and with lower proportions of people in more deprived socioeconomic quintiles. The majority of participants had either no diabetic retinopathy or mild diabetic retinopathy at baseline. There were no significant differences in baseline diabetic retinopathy gradings between CSII vs MDI cohorts.

**Table 1:** Baseline demographics and metabolic characteristics

Characteristic	CSII (*n*=204)	MDI (*n*=211)	*p* value
Age at diagnosis (years)	15 (10–26)	21 (12–33)	<0.001
Age at study entry (years)	38 (29–48)	43 (31–53)	<0.001
Diabetes duration (years)	18 (11–25)	16 (8–27)	0.63
Sex M:F (%M)	76:128 (37.3)	90:121 (42.7)	0.31
Weight (kg)	78.9 (67.6–90.3)	79.5 (67.6–91.5)	0.84
BMI (kg/m^2^)	26.9 (24.0–31.2)	27.1 (24.2–30.4)	0.97
HbA_1c_ (mmol/mol)	66 (58–74)	67 (60–80)	<0.05
HbA_1c_ (%)	8.2 (7.5–8.9)	8.3 (7.6–9.5)	<0.05
SIMD	4 (3–5)	3 (2–5)	<0.05
Cholesterol (mmol/l)	4.7 (4.2–5.2)	4.8 (4.2–5.4)	0.14
Creatinine (μmol/l)	73 (67–83)	74 (68–84)	0.26
Systolic BP (mm/Hg)	128 (118–140)	128 (120–138)	0.95
Diastolic BP (mm/Hg)	78 (73–84)	77 (71–83)	<0.001
Smoking status			0.20
Non-smoker	141 (69)	131 (62)	
Ex-smoker	48 (24)	55 (26)	
Current smoker	15 (7)	25 (12)	
Baseline diabetic retinopathy grading			0.10
R0	93 (45.6)	100 (47.4)	
R1	109 (53.4)	101 (47.9)	
R2	0	2 (0.9)	
R3	2 (1.0)	8 (3.8)	
R4	Excluded	Excluded	

Data are median (IQR) or number (%) unless otherwise indicated. *p* values were calculated using unpaired *t* test for continuous numerical data and χ^2^ test or Fisher’s exact test for categorical data. Baseline grading indicates the overall baseline retinopathy grading for each individual. As retinal data were available for both eyes, the more severe retinal grading outcome for that individual was used as the baseline

F, female; M, male

### HbA_1c_ changes in CSII and MDI

The median number of 6-monthly HbA_1c_ values per person collected over a 3 year study interval was 5 (IQR 4–6). There was a small but statistically significant difference in baseline HbA_1c_ (CSII 66 mmol/mol [8.2%] vs MDI 67 mmol/mol [8.3%], *p* < 0.01). In the CSII group, there were significant reductions in mean study HbA_1c_ (62 mmol/mol [7.8%]), 1 year HbA_1c_ (59 mmol/mol [7.5%]) and study exit HbA_1c_ (61 mmol/mol [7.7%]) from baseline (all *p* < 0.001). In contrast, there were no significant changes in mean study HbA_1c_, 1 year HbA_1c_ or study exit HbA_1c_ (all 67 mmol/mol [8.3%]) from baseline in the MDI group. At all follow-up timepoints, CSII participants had a significantly lower HbA_1c_ than MDI participants (*p* < 0.001), and a greater HbA_1c_ reduction (1 year: −6 mmol/mol [−0.6%] vs −1 mmol/mol [−0.1%], *p* < 0.01; study exit: −4 mmol/mol [−0.4%] vs −2 mmol/mol [−0.2%], *p* = 0.12). In both groups, univariate regression analysis showed higher baseline HbA_1c_ was significantly associated with greater reductions in HbA_1c_ at 1 year (*p* < 0.0001).

The distribution of HbA_1c_ in the CSII and MDI groups is shown in Table 2. There was a higher proportion of MDI vs CSII participants in the highest baseline HbA_1c_ group (>75 mmol/mol [>9%]) but differences were not statistically significant (Table 2). Follow-up HbA_1c_ in CSII and MDI groups at 1 year increased by 5 mmol/mol (0.5%) in 11.1% vs 18.1% and decreased by more than 5 mmol/mol (0.5%) in 51.9% vs 29.3% (*p* < 0.001; Table 2). Changes at study exit showed a similar pattern between MDI and CSII groups, though numbers of participants with available data for analysis were lower (*p* = 0.03; Table 2).

**Table 2:** Stratified HbA_1c_ analysis in CSII and MDI groups

Variable	CSII (*n*=204)	MDI (*n*=211)	*p* value
Baseline HbA_1c_ (/total *n* with data)	/193	/209	0.06
<58 mmol/mol (<7.5%), *n* (%)	42 (21.8)	44 (21.1)	
58–75 mmol/mol (7.5–9%), *n* (%)	107 (55.4)	96 (45.9)	
>75 mmol/mol (>9%), *n* (%)	44 (22.8)	69 (33)	
Change in HbA_1c_ at 1 year (/total *n* with data)	/189	/188	<0.001
>−5 mmol/mol (>−0.5%), *n* (%)	98 (51.9)	55 (29.3)	
−5 to 5 mmol/mol (−0.5 to 0.5%), *n* (%)	70 (37.0)	99 (52.7)	
>5 mmol/mol (>0.5%), *n* (%)	21 (11.1)	34 (18.1)	
Change in HbA_1c_ at study exit (/total *n* with data)	/142	/124	0.03
>−5 mmol/mol (>−0.5%), *n* (%)	63 (44.4)	36 (29.0)	
−5 to 5 mmol/mol (−0.5 to 0.5%), *n* (%)	58 (40.8)	61 (49.2)	
>5 mmol/mol (>0.5%), *n* (%)	21 (14.8)	27 (21.8)	

Table showing numbers of all participants stratified to subgroups for baseline HbA_1c_ and change in HbA_1c_ at 1 year and study exit. *p* values comparing subgroups for all CSII and MDI or matched CSII and MDI cohorts were calculated using χ^2^ test

### Retinopathy events in CSII vs MDI

**Fig. 2:**
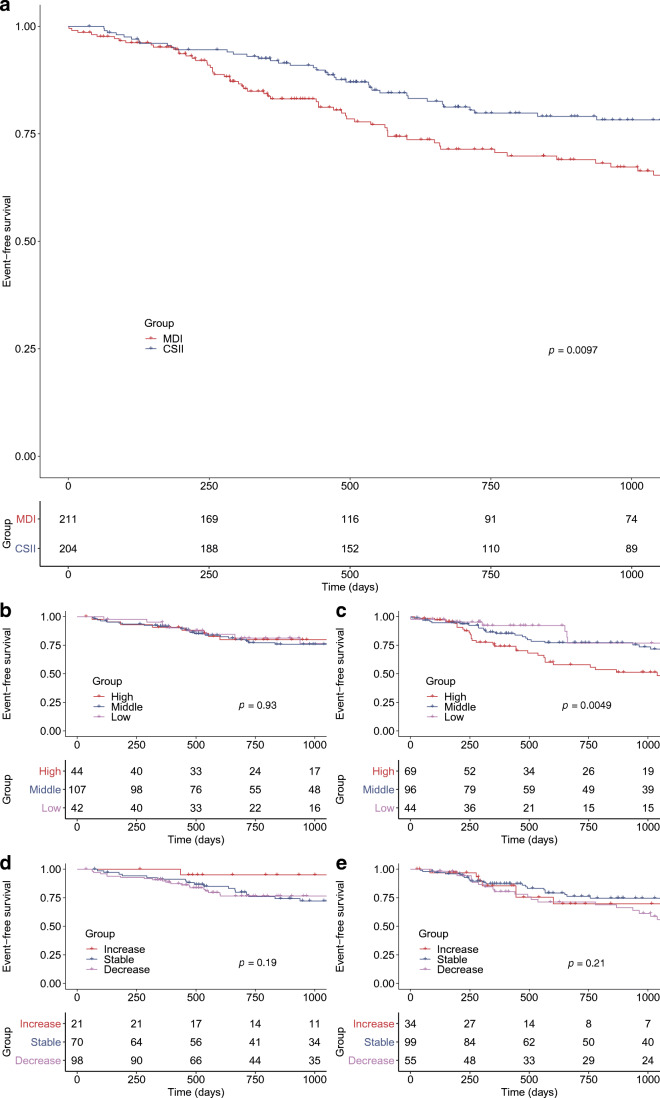
Retinopathy in comparator treatment groups (**a**) and HbA_1c_ subgroups (**b**–**e**). Kaplan–Meier survival plots compare event-free survival rates in CSII/MDI treatment groups (**a**) and HbA_1c_ subgroups (**b**–**e**). An event corresponds to diabetic retinopathy progression and was defined as a minimum one-grade worsening in either eye from the baseline grading. Vertical dashes indicate participants who were censored due to incomplete 3 year follow-up. (**a**) Comparison of CSII (blue) and MDI (red) participants for the entire cohort. CSII was associated with significantly reduced retinopathy progression over 3 years compared with MDI (*p*=0.0097). (**b**, **c**) Comparison of participants with baseline HbA_1c_ <58 mmol/mol (7.5%) (low: lilac), 58–75 mmol/mol (7.5–9%) (middle: blue) or >75 mmol/mol (9%) (high: red) in the CSII group (**b**) and the MDI group (**c**). High baseline HbA_1c_ (>75 mmol/mol [9%]) was associated with increased diabetic retinopathy progression in the MDI group (*p*=0.0049) but was not a determinant of diabetic retinopathy progression in the CSII group (*p*=0.93). (**d**, **e**) Comparison of unmatched participants with change in HbA_1c_ at 1 year of less than −5 mmol/mol (0.5%) (decrease: lilac), −5 to 5 mmol/mol (0.5 to 0.5%) (stable: blue) or more than 5 mmol/mol (0.5%) (increase: red) in the CSII group (**d**) and the MDI group (**e**). Change in HbA_1c_ at follow-up did not significantly impact diabetic retinopathy progression in either cohort

Kaplan–Meier curves describing time to a diabetic retinopathy progression event for comparator groups are shown in Fig. 2a–e. Mean follow-up for the entire cohort was 839 days (2.3 years), with diabetic retinopathy progression occurring in 38 participants (18.6%) in the CSII group vs 56 participants (26.5%) in the MDI group (Fig. 2a; *p* = 0.0097). Of these, unilateral retinal progression was identified in 21/56 (37.5%) people in the MDI group vs 14/38 (36.8%) people in the CSII group, while the rest had bilateral retinal progression.

Pump participants had a reduced risk of diabetic retinopathy progression on univariate (HR 0.58, *p* = 0.01) and multivariate (HR 0.56, *p* = 0.02) Cox proportional hazards analysis, adjusting for age, sex, diabetes duration, baseline HbA_1c_, BP, cholesterol, creatinine, SIMD, smoking status and baseline diabetic retinopathy grading (Table 3).

**Table 3:** Hazard ratios for covariates on univariate and multivariate Cox proportional hazards analysis

Covariate	Univariate analysis			Multivariate analysis		
	HR	(95% CI)	*p* value	HR	(95% CI)	*p* value
Pump group	0.58	0.39, 0.88	0.01	0.56	0.34, 0.91	0.02
Age at study entry	0.98	0.96, 0.99	0.99	0.97	0.96, 0.99	<0.01
Male sex	0.84	0.55, 1.29	0.43	0.69	0.40, 1.19	0.18
Diabetes duration	1.00	0.98, 1.01	0.65	1.03	1.02, 1.05	<0.001
Baseline HbA_1c_	1.03	1.01, 1.03	<0.001	1.03	1.01, 1.04	<0.001
Systolic	1.00	0.98, 1.01	0.68	1.00	0.98, 1.02	0.92
Diastolic	0.99	0.97, 1.02	0.61	1.01	0.99, 1.04	0.36
Cholesterol	0.97	0.78, 1.22	0.82	0.94	0.73, 1.21	0.62
Creatinine	1.00	0.99, 1.01	0.87	1.01	1.00, 1.02	0.01
SIMD	0.89	0.77, 1.03	0.13	0.94	0.79, 1.12	0.51
Smoking status						
Non-smoker	1.00 (Ref.)			1.00 (Ref.)		
Ex-smoker	1.54	0.98, 2.41	0.06	1.89	1.13, 3.17	0.02
Current smoker	1.60	0.84, 3.08	0.15	0.94	0.43, 2.07	0.88
Baseline diabetic retinopathy grading						
R0	1.00 (Ref.)			1.00 (Ref.)		
R1	0.11	0.06, 0.20	<0.001	0.07	0.03, 0.13	<0.001
R2	5.43	1.32, 22.32	0.02	2.90	0.62, 13.6	0.18
R3	1.04	0.38, 2.84	0.94	0.28	0.09, 0.86	0.03

Ref., reference

Those with mild retinopathy at baseline were also at low risk of progression (HR 0.07), with the majority of events occurring in those with no baseline retinopathy (76/94, 80.9%). Older age was also associated with reduced risk of diabetic retinopathy progression (HR 0.97), though longer diabetes duration (HR 1.03), increased creatinine (HR 1.01) and history of previous smoking (HR 1.89) were associated with increased diabetic retinopathy progression risk on multivariate analysis.

Higher baseline HbA_1c_ was associated with increased diabetic retinopathy progression risk for the entire cohort on univariate and multivariate analyses (HR 1.03, *p* < 0.001; Table 3). However, this effect was driven by the MDI cohort, with baseline HbA_1c_ having no significant impact on the frequency of diabetic retinopathy progression occurring in the CSII cohort (Fig. 2b; *p* = 0.93), while in the MDI cohort HbA_1c_ >75 mmol/mol (>9%) was associated with a significantly higher proportion of participants with diabetic retinopathy progression (28/69 participants, 40.6%) than those with HbA_1c_ <58 mmol/mol (<7.5%) (6/44 participants, 13.6%) (Fig. 2c; *p* = 0.0049). The same outcomes were found when groups were stratified by mean study HbA_1c_, with no significant impact of high mean study HbA_1c_ on diabetic retinopathy progression noted in the CSII cohort (*p* = 0.26), while high mean study HbA_1c_ was associated with increased diabetic retinopathy progression in the MDI cohort (*p* = 0.023). HbA_1c_ values at 1 year and study exit showed no significant impact on the frequency of diabetic retinopathy progression in either cohort. Change in HbA_1c_ at 1 year from baseline was not associated with diabetic retinopathy progression in either cohort (CSII: Fig. 2d [*p* = 0.19], MDI: Fig. 2e [*p* = 0.21]).

## Discussion

In this real-world study, using a robust clinical database and a nationalised single diabetic retinopathy scoring system, we observed that CSII therapy was associated with significantly lower diabetic retinopathy progression over a 3 year follow-up period in adults with type 1 diabetes who had completed structured diabetes education than in those on MDI therapy who had completed an equivalent structured diabetes education programme (18.6% in CSII vs 26.5% in MDI, *p* = 0.0097). In addition, there was no evidence of early diabetic retinopathy worsening in those using CSII compared with MDI over the first 18 months. This is despite significantly larger reductions in HbA_1c_ in the CSII group (6 mmol/mol [0.5%] reduction) vs the MDI group (1 mmol/mol [0.1%] reduction) at 1 year post study entry (*p* < 0.001). Findings were confirmed on multivariate analysis adjusting for multiple potential confounders.

Those with longer duration of diabetes were found to be at higher risk of diabetic retinopathy progression, which was expected as this is a well-established risk factor for diabetic retinopathy [1]. Surprisingly, older age was associated with a small but significant reduction in risk of diabetic retinopathy progression in our cohort. This was driven by MDI participants, with no significant effect of age on diabetic retinopathy progression when CSII participants were analysed in isolation. The addition of drug therapies such as statins and antihypertensives in older participants may have contributed to diabetic retinopathy risk reduction; however, we were unable to assess this in our cohort.

Studies of diabetic retinopathy progression in adults are lacking in real-world settings. However, our findings are consistent with a longitudinal study of adolescents (aged 12–20 years) treated with either CSII or MDI therapy over a period of 15 years [9]. The study showed a significantly lower risk of developing retinopathy in those treated with CSII vs MDI (OR 0.66), though the majority of assessments (79%) were from participants who were only reviewed once during the study period. Proportions of people developing retinopathy were slightly lower than in our cohort (17% in CSII, 22% in MDI); however, this is likely to be consistent with a patient cohort of younger age and shorter diabetes duration than in our study. A further study assessed retinal changes in 31 adults following initiation of CSII using a range of imaging modalities, including OCT, and showed stable retinal characteristics over 1 year with no evidence of early diabetic retinopathy worsening [12].

High baseline HbA_1c_ (75 mmol/mol [>9%]) was associated with increased diabetic retinopathy progression in the MDI group. In contrast, diabetic retinopathy progression in the CSII group was not found to be associated with baseline HbA_1c_. In the MDI group there were no significant reductions in follow-up HbA_1c_ values when compared with baseline levels. However, in the CSII group, follow-up HbA_1c_ values were significantly reduced from baseline, and were lower than HbA_1c_ levels assessed at the same timepoints in the MDI group. Absolute change in HbA_1c_ at 1 year from baseline was not significantly associated with diabetic retinopathy progression for either group.

We hypothesise that factors intrinsic to CSII therapy are protective against diabetic retinopathy progression in those with high baseline HbA_1c_, while those with high baseline HbA_1c_ on MDI remain at increased diabetic retinopathy risk. Many of the MDI participants did not achieve a substantial reduction in HbA_1c_ on follow-up and therefore had continued exposure to high glycaemic levels which may have contributed to increased diabetic retinopathy risk. This is supported by the fact that high mean study HbA_1c_ was significantly associated with diabetic retinopathy progression in the MDI group, though this was not true in the CSII group.

While exposure to high glycaemic levels is a well-established risk factor for diabetic retinopathy, recurrent disabling hypoglycaemia may also increase diabetic retinopathy risk [13]. Though recurrent disabling hypoglycaemia is a possible indication for consideration of CSII therapy, it was not possible for this to be accurately assessed within our cohorts and levels of hypoglycaemia within the groups may have differed. It is therefore possible that some of those perceived to have good glycaemic control, reflected by a lower baseline HbA_1c_, were exposed to higher levels of hypoglycaemia prior to the study period, which could have contributed to increased diabetic retinopathy risk. CSII therapy has been shown to reduce frequency of hypoglycaemia [14], which may also have conferred a benefit in terms of diabetic retinopathy risk, even in those with no HbA_1c_ reduction.

Other factors related to better control of diabetes (including glycaemic variability [GV], discussed further below) may also have contributed to the reduction in diabetic retinopathy observed in the CSII group.

It is important to note that, within our retinopathy screening programme in Scotland, when people are commenced on CSII therapy, recommendations are in place with regard to modifications to early HbA_1c_ targets where appropriate, particularly for those with high baseline HbA_1c_ levels, and improved surveillance for retinopathy [5]. As such, the reductions in HbA_1c_, although significant in our study, may have been achieved more gradually in the CSII group, and may have facilitated improved retinopathy outcomes.

Glycaemic control as measured by HbA_1c_ has been shown to be important in reducing diabetic retinopathy progression, as evidenced from the DCCT study [15]. However, other studies do not demonstrate an association between baseline HbA_1c_, or change in HbA_1c_, and diabetic retinopathy progression following initiation of CSII therapy [16]. Evidence from the DCCT also showed that HbA_1c_ changes did not fully explain the risk of diabetic retinopathy progression in type 1 diabetes, and that other features of glucose control, such as the extent of postprandial glucose excursions or counterregulatory responses to hypoglycaemia, which are not easily reflected by a summary measure such as HbA_1c_, may have an impact on the risk of developing complications [15]. DCCT analyses assessing time in range (TIR) from seven-point fingerstick data showed this had a strong association with the development of microvascular complications including retinopathy [17], though earlier DCCT analyses suggest that within-day GV and mean amplitude of glucose excursions (MAGE) were not predictive of diabetic retinopathy [18].

More widespread availability of continuous glucose monitoring (CGM) in recent years has led to expanding interest in the role of glycaemic markers other than HbA_1c_, including TIR, GV and MAGE, with recommendations now in place for their use in routine diabetes management [19].

There is growing evidence from CGM that these markers are associated with diabetic retinopathy in type 2 diabetes [20–22]; however, less is known about their role in type 1 diabetes [23]. We and others have reported that islet transplantation in recipients with type 1 diabetes is associated with diminished GV and reductions in HbA_1c_ in association with diminished progression of diabetic retinopathy [24, 25, unpublished data (Reid L, Lam A, Dhillon B, Duncan K, Ibbotson C, Sutherland A, Casey J, Koh A, Rudinsky C, Tennant M, Malcolm A, Shapiro AMJ, Senior P, Forbes S (2019)]. Further studies assessing the impact of GV on diabetic retinopathy progression are needed.

CSII is associated with higher initial costs due to the expense of the pump, necessary consumables and pump education [26]. However, longer-term cost–benefit analyses studies indicate that such costs are offset by improved glycaemic control, enhanced quality-of-life markers and reductions in diabetes complications, and demonstrate that CSII is in fact a cost-effective treatment in type 1 diabetes [26, 27]. Many cost–benefit models highlight the reduced morbidity- and mortality-related costs secondary to reductions in problematic hypoglycaemia associated with CSII therapy [26], and projected cost savings related to diminished micro- and macrovascular complications are also evident, with an 18.3% lifetime reduction in severe visual loss with CSII vs MDI therapy [27].

The strengths of this study are that it is a relatively large study providing real-world data on diabetic retinopathy progression in type 1 diabetes in an adult cohort following initiation of CSII therapy. Furthermore, diabetic retinopathy assessment was performed within a single retinopathy screening programme. It provides reassurance that, in people with no diabetic retinopathy or mild diabetic retinopathy at baseline, there is no evidence of increased risk of diabetic retinopathy progression following initiation of CSII, and there is long-term benefit particularly in those with the highest HbA_1c_ compared with continued MDI therapy.

The study has several limitations. As a retrospective real-world study, timings of retinal imaging and HbA_1c_ collection were not uniform for participants over the study period. In addition, participants were not randomised to receive either CSII or MDI therapy, resulting in potential confounding and allocation bias. Indeed, at baseline the CSII group was significantly younger with earlier age of diabetes onset, lower baseline HbA_1c_ and lower diastolic BP, and lived in less socioeconomically deprived areas than the MDI group. Multivariate analyses were performed to minimise the impact of these differences. Study entry time for the MDI group, who did not have any specific intervention, was based on the lag time from receiving structured education to commencing CSII therapy. Although there was some variability in this lag time, when data were reassessed using an alternative study entry time based on the median lag time (841 days) rather than mean lag time (945 days) (see ESM Methods), outcomes were unchanged, with results still showing significantly fewer retinal events in the CSII group vs the MDI group (see ESM Results and ESM Fig. 1). Retinal data were sourced from the diabetes retinopathy screening programme, and therefore people who had no retinal screening results could not be assessed (7.8% CSII, 12.3% MDI). This could represent a population of non-attenders who are not fully engaging with aspects of their diabetes management, and as such may be at higher risk of developing diabetic retinopathy. In addition, few participants with advanced retinal disease, who are known to be at the highest level of risk of diabetic retinopathy [28], were assessed as these people leave the screening programme to enter into a management pathway under the care of ophthalmologists, though numbers excluded due to receiving ophthalmology care were similar in both groups (10.6% CSII, 10.1% MDI). Reporting of retinopathy outcomes using SDRGS assessment is not the gold standard, and therefore we may have missed more subtle retinal changes that could have been picked up by using Early Treatment Diabetic Retinopathy Study (ETDRS) screening; however, as SDRGS is designed to detect clinically significant changes we feel findings are still clinically relevant. These problems relating to the use of screening data will have affected both groups, and therefore their impact on the study analysis was felt to be relatively small. Glycaemic analysis was limited to HbA_1c_ data. Though this remains the gold standard for glycaemic assessment, further studies using CGM will help fully characterise the range of glycaemic factors involved in diabetic retinopathy progression. Finally, this study was conducted in a predominantly white population across three diabetes centres from a single Scottish region, where a national diabetic retinopathy screening service is offered for all people affected by diabetes. Therefore, results may not be generalisable to all people with type 1 diabetes from other ethnic groups, or where access to diabetic retinopathy screening is limited.

### Conclusion

This observational study has demonstrated reduced diabetic retinopathy progression in a real-world population with type 1 diabetes commenced on CSII vs those who continued on MDI, with no evidence of early diabetic retinopathy worsening. Although reductions in HbA_1c_ were seen due to CSII therapy, these were not associated with a reduction in diabetic retinopathy progression and as such other glycaemic factors associated with CSII therapy may play a role. Further prospective studies using CGM may help establish if reductions in glycaemic excursions are causal in reducing the progression of diabetic retinopathy and other microvascular diabetes complications.

## Supplementary information

ESM (PDF 158 kb)

## Data Availability

The datasets generated during and/or analysed during the current study are available from the corresponding author on reasonable request.

## Abbreviations

CGM: Continuous glucose monitoring
CSII: Continuous subcutaneous insulin infusion
GV: Glycaemic variability
MAGE: Mean amplitude of glucose excursions
MDI: Multiple daily insulin injections
OCT: Ocular coherence tomography
SCI-Diabetes: Scottish Care Information – Diabetes
SDRGS: Scottish Diabetic Retinopathy Grading Scheme
SIMD: Scottish Index of Multiple Deprivation
TIR: Time in range
