# Supplementary material for: Continuous subcutaneous insulin infusion therapy is associated with reduced retinopathy progression compared with multiple daily injections of insulin
Source: Diabetologia. 2021 May 8;64(8):1725–36. doi: 10.1007/s00125-021-05456-w (PMC8245368; doi:10.1007/s00125-021-05456-w)

Electronic Supplementary Material (ESM)

ESM Methods

A secondary analysis was carried out using an alternative study entry for the MDI group. This was calculated using the median lag time from education to CSII commencement, rather than the mean lag time which was used for the main analysis. This was equivalent to 841 days from the date of completion of the diabetes education course. The remainder of the methodology was unchanged from the main analysis.

ESM Results

Three people who were excluded in the primary analysis due to lack of follow up retinal data were able to be included following the shift in study entry time, bringing the total number of people included in the MDI group to 214.

The number of events in MDI group was 55, versus 38 events in CSII group which were unchanged. This remained significant on univariate ($p=0.03$) and multivariate ($p=0.045$) analyses, with no evidence of early DR worsening in the CSII group. The updated survival curve is also shown below (ESM Figure 1).

ESM Figure 1: Survival curve using study entry for the MDI group as 841 days from the structured education course, based on median lag time from the education to commencement of CSII therapy:

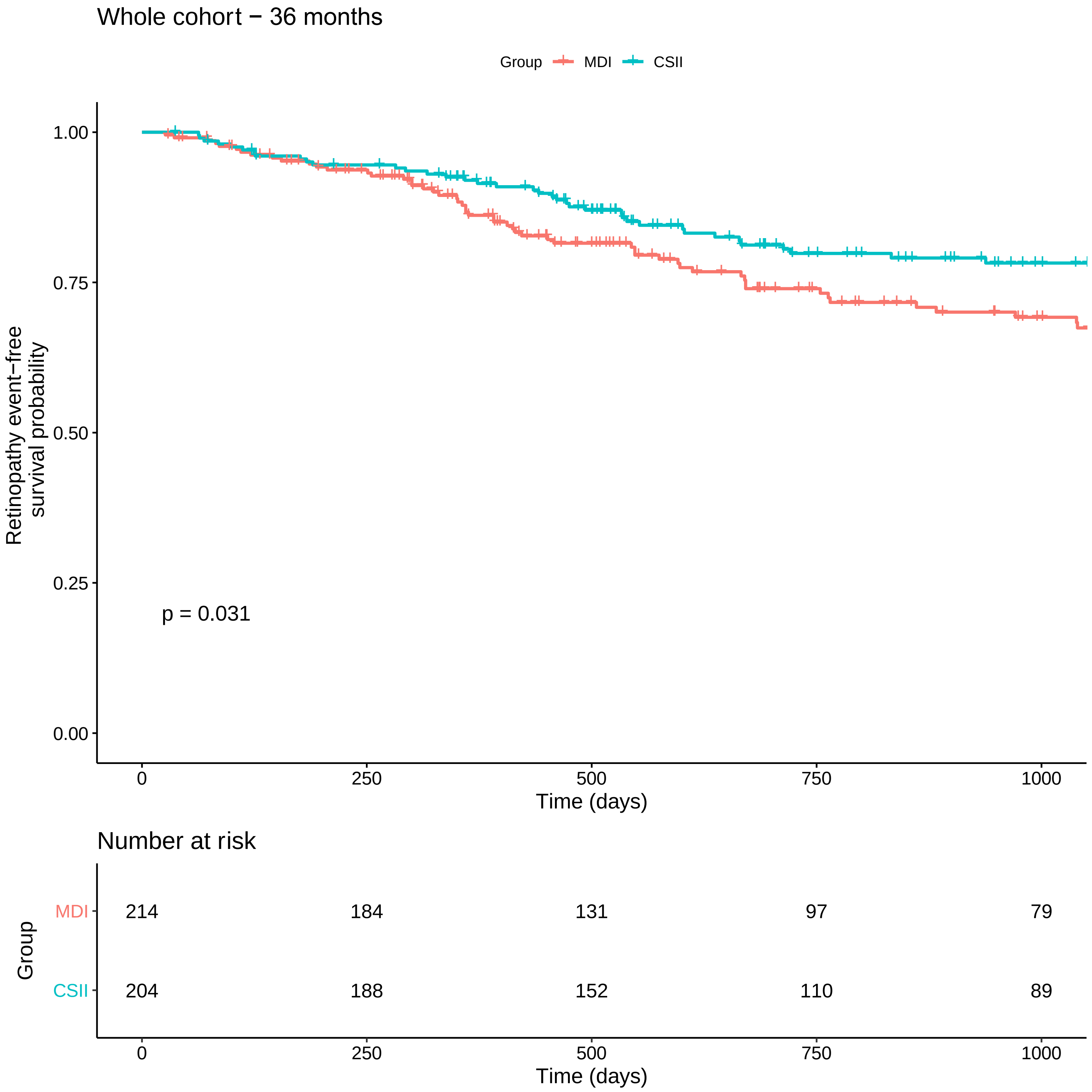

Supplement: Supplementary file 1 — (PDF 158 kb) [file 125_2021_5456_MOESM1_ESM.pdf]
